# Non-invasive prediction for pathologic complete response to neoadjuvant chemoimmunotherapy in lung cancer using CT-based deep learning: a multicenter study

**DOI:** 10.3389/fimmu.2024.1327779

**Published:** 2024-03-25

**Authors:** Wendong Qu, Cheng Chen, Chuang Cai, Ming Gong, Qian Luo, Yongxiang Song, Minglei Yang, Min Shi

**Affiliations:** ^1^ Department of Thoracic Surgery, Affiliated Hospital of Zunyi Medical University, Zunyi, China; ^2^ School of Computer Science and Communication Engineering, Jiangsu University, Zhenjiang, Jiangsu, China; ^3^ Department of Thoracic Surgery, Ningbo Hwamei Hospital, Chinese Academy of Sciences, Zhejiang, China; ^4^ Department of Oncology, Ruijin Hospital, Shanghai Jiao Tong University School of Medicine, Shanghai, China; ^5^ Shanghai Hospital of Civil Aviation Administration of China, Shanghai, China; ^6^ Department of Oncology, Wuxi Branch of Ruijin Hospital, Shanghai Jiao Tong University School of Medicine, Wuxi, China

**Keywords:** deep learning, neoadjuvant chemoimmunotherapy, pathologic complete response, lung cancer, radiomics

## Abstract

Neoadjuvant chemoimmunotherapy has revolutionized the therapeutic strategy for non-small cell lung cancer (NSCLC), and identifying candidates likely responding to this advanced treatment is of important clinical significance. The current multi-institutional study aims to develop a deep learning model to predict pathologic complete response (pCR) to neoadjuvant immunotherapy in NSCLC based on computed tomography (CT) imaging and further prob the biologic foundation of the proposed deep learning signature. A total of 248 participants administrated with neoadjuvant immunotherapy followed by surgery for NSCLC at Ruijin Hospital, Ningbo Hwamei Hospital, and Affiliated Hospital of Zunyi Medical University from January 2019 to September 2023 were enrolled. The imaging data within 2 weeks prior to neoadjuvant chemoimmunotherapy were retrospectively extracted. Patients from Ruijin Hospital were grouped as the training set (n = 104) and the validation set (n = 69) at the 6:4 ratio, and other participants from Ningbo Hwamei Hospital and Affiliated Hospital of Zunyi Medical University served as an external cohort (n = 75). For the entire population, pCR was obtained in 29.4% (n = 73) of cases. The areas under the curve (AUCs) of our deep learning signature for pCR prediction were 0.775 (95% confidence interval [CI]: 0.649 - 0.901) and 0.743 (95% CI: 0.618 - 0.869) in the validation set and the external cohort, significantly superior than 0.579 (95% CI: 0.468 - 0.689) and 0.569 (95% CI: 0.454 - 0.683) of the clinical model. Furthermore, higher deep learning scores correlated to the upregulation for pathways of cell metabolism and more antitumor immune infiltration in microenvironment. Our developed deep learning model is capable of predicting pCR to neoadjuvant chemoimmunotherapy in patients with NSCLC.

## Introduction

Surgical excision remains the cornerstone of curative treatment for non-small-cell lung cancer (NSCLC) at early and locally advanced stages (1). Nevertheless, despite achieving a radical removal, disease controls remain disheartening, with postsurgical relapses observed in 30% to 60% of cases (2). Furthermore, an inclusion of chemotherapy at neoadjuvant or adjuvant context provides marginal advantages for decreasing recurrent rates, resulting in a mere 5% improvement in overall survival (3), this underscores the imperative for innovative approaches in managing resectable lung cancer. In recent years, immunotherapy, specifically immune checkpoint inhibitors (ICIs), presented remarkable antitumor efficacy, fundamentally reshaping the therapeutic landscape for lung cancer (4). This offers a compelling rationale for adopting neoadjuvant immunotherapy for resectable lung cancer.

The effectiveness of neoadjuvant chemoimmunotherapy has been thoroughly explored in numerous studies (5–8), indicating that it has the potential to reduce the tumor burden before surgical resection, thereby decreasing the risks of recurrence. This approach unveils promising avenues for resectable NSCLC treatment. However, a significant proportion of patients do not attain pathologic complete response (pCR) to neoadjuvant immunotherapy (9), underscoring the urgent and unmet need to identify NSCLC patients who might positively respond to this therapeutic strategy.

Existing evidence suggests that various biomarkers, including tumor mutation burden (10), PD-L1 expressions (11), tumor-infiltrating lymphocytes (4) and inflammatory cytokines (12), are linked to the response to ICIs. However, the identification of these biomarkers relies primarily on biopsy, which carries a significant morbidity risk due to its invasive nature and cannot capture the tumor’s full heterogeneity due to the small specimens (13, 14). Consequently, developing a robust and non-invasive biomarker for predicting pCR to neoadjuvant immunotherapy in lung cancer is of utmost significance.

Deep learning, with a capability to quantify high-throughput radiomics features which elude human perception then directly develop corresponding prediction signatures for diverse clinical scenarios (15), provides a non-invasive tool for tumor diagnosis (16), treatment decisions (17) and survival estimations (18). Prior studies have illuminated the associations of radiomics features with the response to neoadjuvant chemotherapy in resectable NSCLC (19, 20) and the prognosis of immunotherapy in advanced NSCLC (21, 22). However, limited evidence currently supports the efficacy of the deep learning algorithm to estimate responses to neoadjuvant immunotherapy for lung cancer. In this perspective, this study, rooted in multi-institutional population, purposes on using computed tomography (CT) imaging to develop a deep learning signature to predict pCR for NSCLC patients undergoing neoadjuvant ICI treatment.

## Methods

### Population and study design

**Figure 1:**
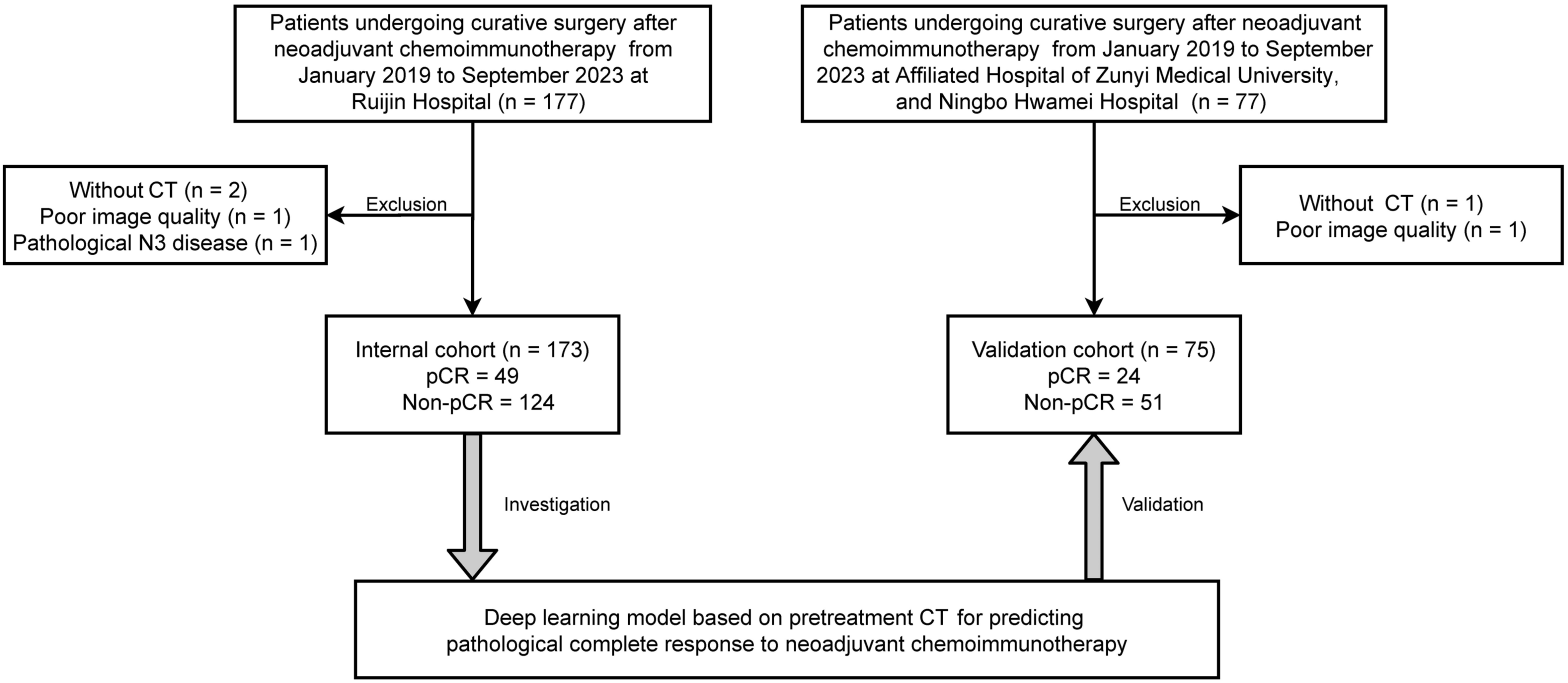
Flowchart illustrates the patient selection.

Included in the current study were patients who underwent neoadjuvant chemoimmunotherapy followed by surgical resection for NSCLC at multiple medical institutions, including Ruijin Hospital, Affiliated Hospital of Zunyi Medical University, and Ningbo Hwamei Hospital, during the period from January 2019 to September 2023 (**Figure 1**). The regimens for neoadjuvant chemoimmunotherapy usually included 2 to 4 cycles of pembrolizumab or nivolumab in combination with platinum-based chemotherapy. We retrospectively gathered baseline information and imaging data taken within 2 weeks prior to the initiation of neoadjuvant treatment.

**Figure 2:**
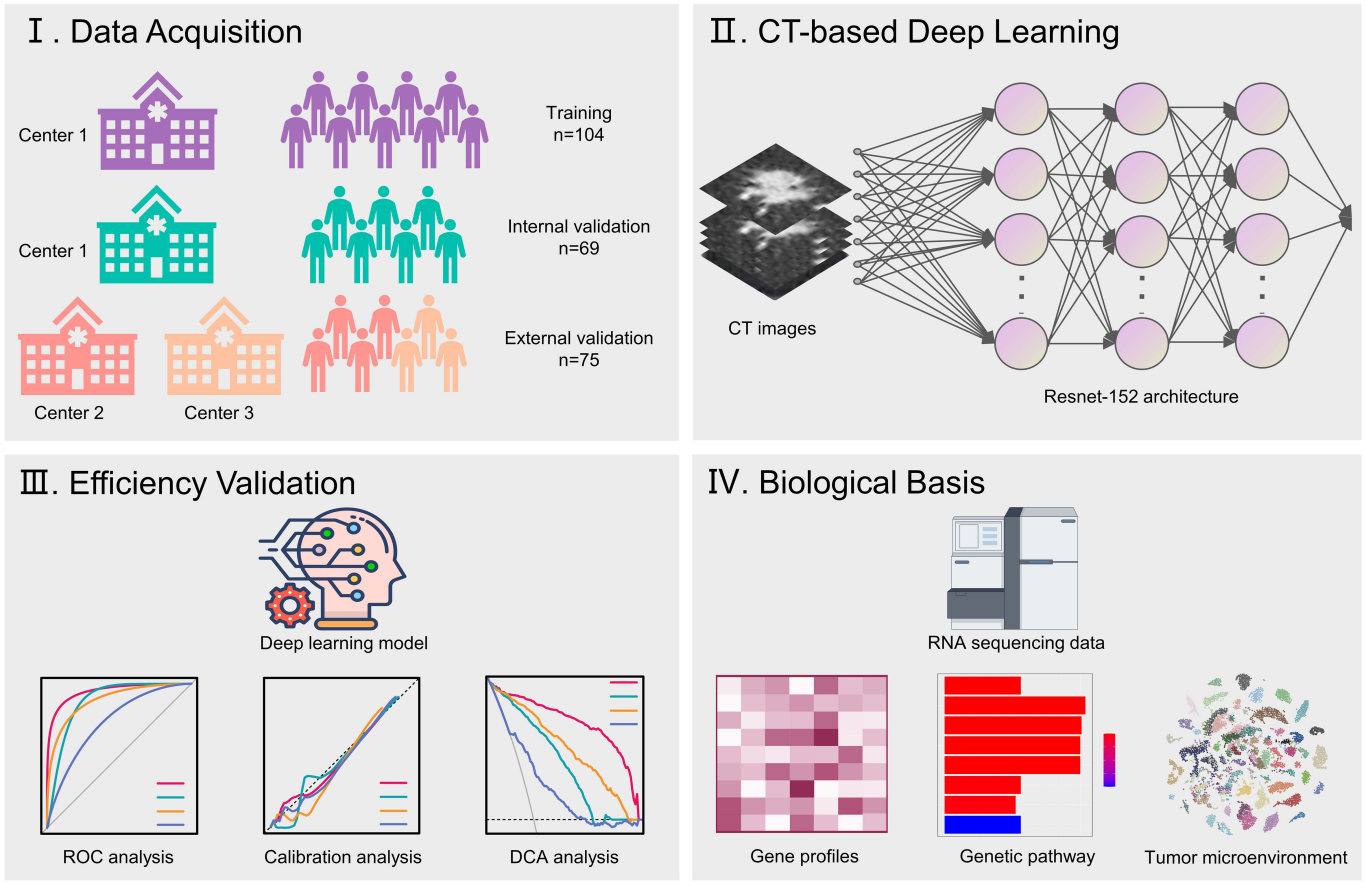
Flowchart illustrates the study design.

The study design was illustrated in **Figure 2**. Patients treated at Ruijin Hospital were grouped as the training set and the validation set with the 6:4 ratio for primarily constructing a CT-based deep learning model. Additionally, all patients treated at Affiliated Hospital of Zunyi Medical University and Ningbo Hwamei Hospital were grouped into an external cohort to validate the proposed deep learning model. Finally, the biological basis of the proposed deep learning model was investigated by analysing genetic pathways and microenvironment infiltrations associated with the deep learning scores.

### Pathologic estimation

Pathologic responses were estimated in accordance with the following standard (23): we firstly calculated the proportions of tumor cell, necrosis and stroma. pCR was estimated to be no viable tumor cell present in primary tumors and lymph nodes. Above procedures were implemented by two pathologists with experiences of more than 10 years independently, if a disagreement arose, discussion was conducted to achieve a consensus.

### Imaging acquisition and tumor segmentation

CT scans were conducted on Somatom Definition AS+ (Siemens Medical Systems, Germany), and iCT256 (Philips Medical Systems, Netherlands). All CT images were resampled as 1 x 1 x 1 mm3 to uniform data and then input into 3D slicer software (www.slicer.org) for annotation. The primary tumor was delineated using a cuboid which encompassed the whole tumor region. Two junior radiologists, each with over 5 years of experience, independently conducted tumor segmentation within a lung window setting (mean, −500HU; width, 1550HU). In instances where interobserver discrepancies arose, they consulted a senior radiologist with experiences of more than 10 years for resolution. Finally, to reduce the batch effect error, pixel-wise CT image data were normalized by z-score normalization.

### Model construction

**Figure 3:**
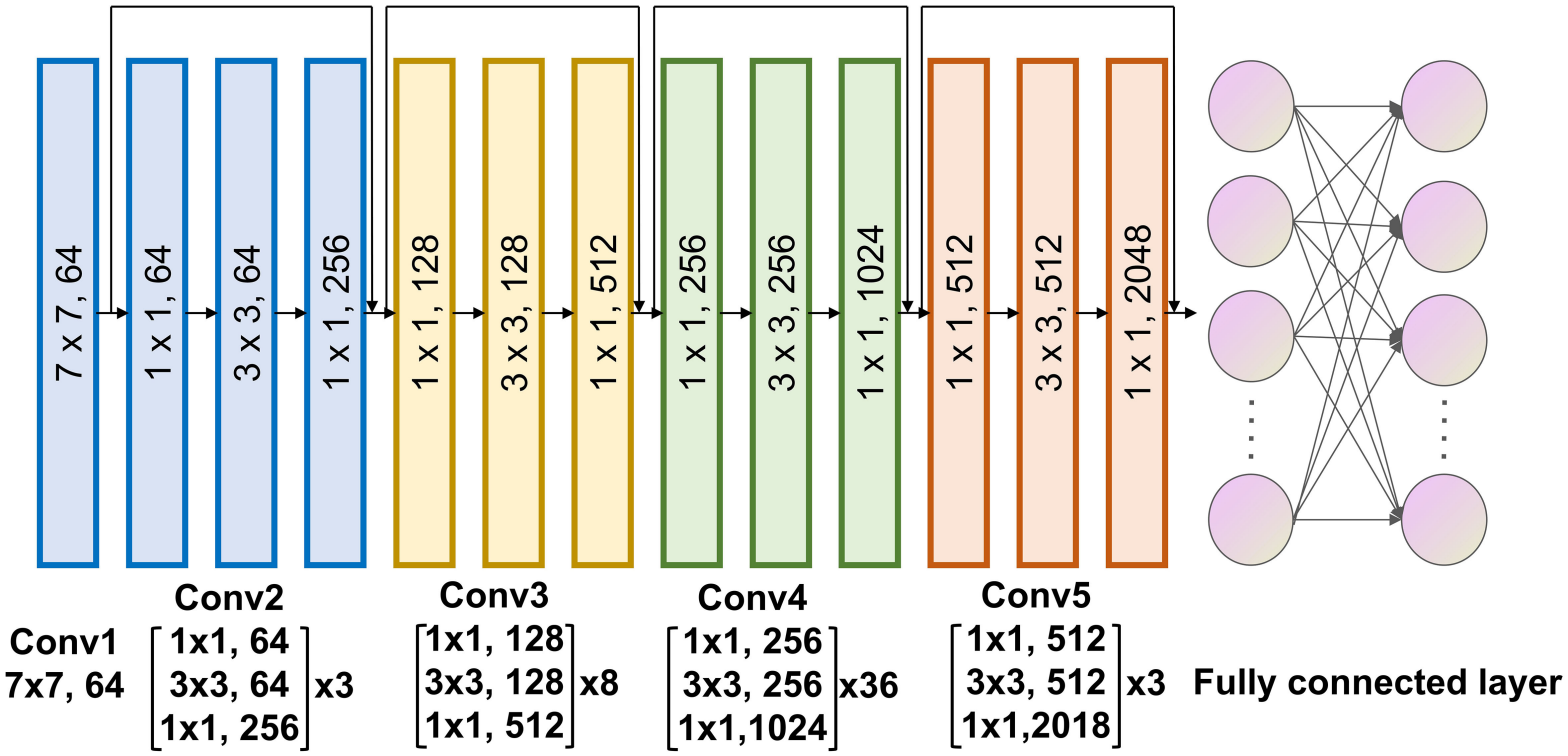
Architecture of the deep learning algorithm.

The deep learning signature to predict pCR to neoadjuvant immunotherapy was constructed using the deep learning algorithm. The final signature comprised a convolutional part and a classification part, was developed at 3 primary steps: Employing a deep learning architecture to serve as a feature extractor. Connecting it to a fully connected layer to further quantify high-dimensional phenotypes for precise classification. Unfreezing all network parameters to train the final model using a reduced learning ratio. In terms of the deep learning architecture, we chose to utilize Resnet-152 (**Figure 3**) for feature extraction from CT images of tumor regions. For the fully connected layer, we added 512 nodes prior to the output to enhance the fusion of phenotypes extracted with Resnet-152. Since the Softmax function is incorporated, our model provides direct pCR probability output.

### Biological basis investigation

To elucidate the biologic mechanisms behind the deep learning predictions, we conducted a genetic analysis on a subset of 130 patients from the TCIA dataset who had available RNA-sequencing data. These patients were stratified based on their deep learning scores. We utilized the limma package to determine differentially expressed genes between patients with high and low deep learning score, applying standard of log fold changes more than 2 and adjusted p values less than 0.05.

Following this analysis, we performed Gene Ontology (GO) pathway analyses using the clusterProfiler package to determine pathways associated with the deep learning scores. Additionally, we conducted immune microenvironment analyses using to quantify the proportions of specific immune cells in microenvironment.

### Statistics

The nominal and numerical baseline information was respectively summarized to be frequencies (percentages) and means ± standard deviations. We conducted comparisons via the Chi-square test for nominal data and the t-test for numerical data. For estimating our model’s performance, we primarily plotted receiver operating characteristic curves (ROCs) then computed the corresponding areas under the curve (AUCs). To determine the statistical significance of differences between AUCs, we utilized the DeLong’s test. Univariate and multivariate logistic regression was performed for identifying clinical predictors for pCR and construct clinical model based backward elimination method. Aforementioned statistic procedures were carried out by utilizing R and Python software. A significance level of p < 0.05 was considered as statistically significant.

## Results

### Clinicopathologic information

**Table 1** presents a summary of the baseline information. In total, 248 patients participated in this study, with 104 assigned for training, 69 for internal validation, and 75 for external validation. The overall cohort had a mean age of 61.8 years, with 12.5% (n = 31) of patients being female. Among the cases, 148 (59.7%) were diagnosed as squamous cell carcinomas, while 78 (31.5%) were identified as adenocarcinomas. In terms of pretreatment stage, most participants were staged to be T2 (n = 96, 38.7%) and N2 (n = 167, 67.3%), with stage III (n = 215, 86.7%) being the most common stage in the entire population. Regarding the assessment of pathologic response, 29.4% (n = 73) of patients achieved pCR. No significant differences were observed across the training set, validation set, and external cohort.

**Table 1:** Clinicopathological characteristics of all included patients.

Characteristics	Entire cohort (n = 248)	Internal cohort (n = 173)		External cohort (n = 75)	p1 value	p2 value
		Training (n = 104)	Validation (n = 69)			
Age, Mean ± SD, years	61.8 ± 8.7	61.8 ± 9.7	60.2 ± 8.4	63.1 ± 7.4	0.278	0.334
< 65	137 (55.2)	55 (52.9)	44 (63.8)	38 (50.7)	0.157	0.769
≥65	111 (44.8)	49 (47.1)	25 (36.2)	37 (49.3)		
Gender					0.340	0.074
Female	31 (12.5)	14 (13.5)	13 (18.8)	4 (5.3)		
Male	217 (87.5)	90 (86.5)	56 (81.2)	71 (94.7)		
Smoking status					0.159	0.072
Never	135 (54.4)	64 (61.5)	35 (50.7)	36 (48.0)		
Ever	113 (45.6)	40 (38.5)	34 (49.3)	39 (52.0)		
Pretreatment T					0.153	0.133
T1	35 (14.1)	18 (17.3)	9 (13.0)	8 (10.7)		
T2	96 (38.7)	47 (45.2)	23 (33.3)	26 (34.7)		
T3	60 (24.2)	18 (17.3)	21 (30.4)	21 (28.0)		
T4	57 (23.0)	21 (20.2)	16 (23.2)	20 (26.7)		
Pretreatment N					0.465	0.131
N0	36 (14.5)	13 (12.5)	5 (7.2)	18 (24.0)		
N1	45 (18.2)	18 (17.3)	15 (21.7)	12 (16.0)		
N2	167 (67.3)	73 (70.2)	49 (71.0)	45 (60.0)		
Pretreatment TNM					0.510	0.3911
I	5 (2.0)	2 (1.9)	0 (0)	3 (4.0)		
II	28 (11.3)	10 (9.6)	7 (10.1)	11 (14.7)		
III	215(86.7)	92 (88.5)	62 (89.9)	61 (81.3)		
Histology					0.053	0.333
SCC	148 (59.7)	63 (60.6)	35 (50.7)	50 (66.7)		
Adenocarcinoma	78 (31.5)	36 (34.6)	23 (33.3)	19 (25.3)		
Others	22 (8.9)	5 (4.8)	11 (15.9)	6 (8.0)		
Response					0.125	0.239
pCR	73 (29.4)	25 (24.0)	24 (34.8)	24 (32.0)		
Non-pCR	175 (70.6)	79 (76.0)	45 (65.2)	51 (68.0)		

p1 value for comparisons between the training set and internal validation set; p2 value for comparisons between the training set and external validation cohort; SD, standard deviation; pCR, pathologic complete response; SCC, squamous cell carcinoma.

To prob the clinical predictors for pCR, we implemented the logistic regression for patients at the training set. As presented in **Table 2**, only pretreatment TNM stage (stage II: odds ratio [OR], 0.238; 95% confidence interval [CI], 0.008 – 7.232; p=0.410; stage III: OR, 0.016; 95% CI, 0.001 – 0.961; p=0.048) and histology of adenocarcinoma (OR, 0.309; 95% CI, 0.092 – 0.934; p=0.047) were identified to be significantly associated with pCR.

**Table 2:** Logistic analyses for pathologic complete response in the training set.

Variables	Univariable		Multivariable	
	OR (95% CI)	p value	OR (95% CI)	p value
Age (≥65)	1.985 (0.795 – 4.961)	0.142	2.442 (0.897 – 6.648)	0.081
Gender (Male)	2.060 (0.428 – 9.901)	0.376		
Smoking status (Ever)	0.871 (0.343 – 2.215)	0.772		
Pretreatment T stage				
T1	Reference			
T2	0.686 (0.211 – 2.230)	0.531		
T3	0.571 (0.130 – 2.514)	0.459		
T4	0.333 (0.070 – 1.597)	0.169		
Pretreatment N stage				
N0	Reference			
N1	0.667 (0.111 – 3.990)	0.657		
N2	1.173 (0.292 – 4.719)	0.822		
Pretreatment TNM stage				
I	Reference		Reference	
II	0.667 (0.032 – 14.033)	0.794	0.238 (0.008 – 7.232)	0.410
III	0.278 (0.017 – 4.640)	0.373	0.016 (0.001 – 0.961)	0.048
Histology (Adenocarcinoma)	0.954 (0.388 – 2.349)	0.919	0.309 (0.092 – 0.934)	0.047

OR, odds ratio; CI, confidence interval.

### Efficiency the deep learning model

**Figure 4:**
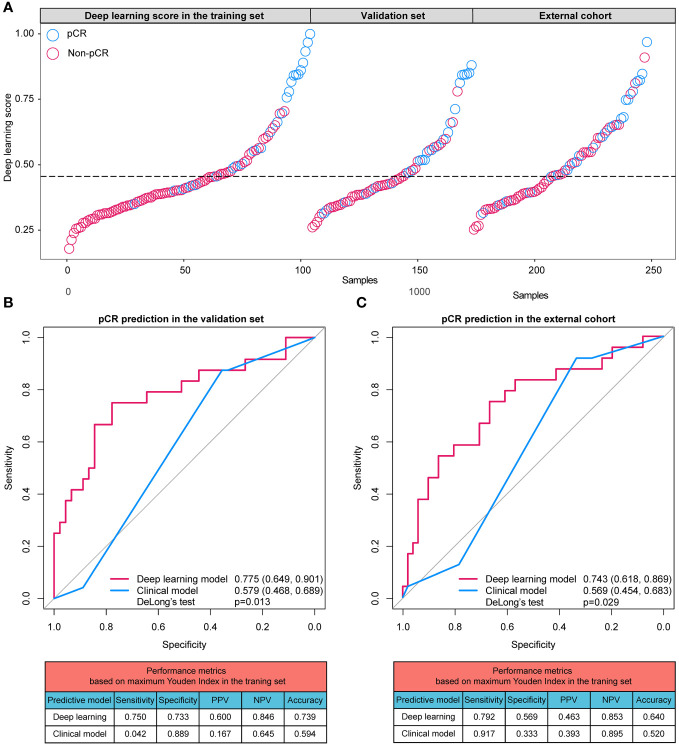
**(A)** Distributions of the deep learning scores in training set, validation set, and external cohort; **(B)** ROC curves and performance metrics of the deep learning model and clinical model in the validation set; **(C)** ROC curves and performance metrics of the deep learning model and clinical model in the external cohort. pCR, pathologic complete response; ROC, receiver operating characteristic curve; PPV, positive predictive value; NPV, negative predictive value.

With an increase of the deep learning score, more participants achieving pCR were identified in all datasets (**Figure 4A**). The AUCs of the deep learning model for distinguishing pCR were 0.775 (95% CI: 0.649 - 0.901) and 0.743 (95% CI: 0.618 - 0.869) in the validation set and external cohort, respectively, which were significantly better than the clinical model (Validation set: 0.579 [95% CI: 0.468, 0.689], p=0.013; External cohort: 0.569 [95% CI: 0.454, 0.683], p=0.029) (**Figures 4B, C**).

Based on the cutoff values calculated based on the training cases, the performance metrics of predictive models were generated. In the validation set, the sensitivity, specificity, positive predictive value (PPV), negative predictive value (NPV) and accuracy of the deep learning model were 0.750, 0.773 0.600, 0.846 and 0.739, respectively, higher than those of the clinical model (sensitivity: 0.042; specificity: 0.889; PPV: 0.167; NPV: 0.645; accuracy: 0.594). Similarly, in the external cohort, the sensitivity, specificity, PPV, NPV and accuracy of the deep learning model were 0.792, 0.569 0.463, 0.853 and 0.640, respectively, higher than those of the clinical model (sensitivity: 0.917; specificity: 0.333; PPV: 0.393; NPV: 0.895; accuracy: 0.520).

**Figure 5:**
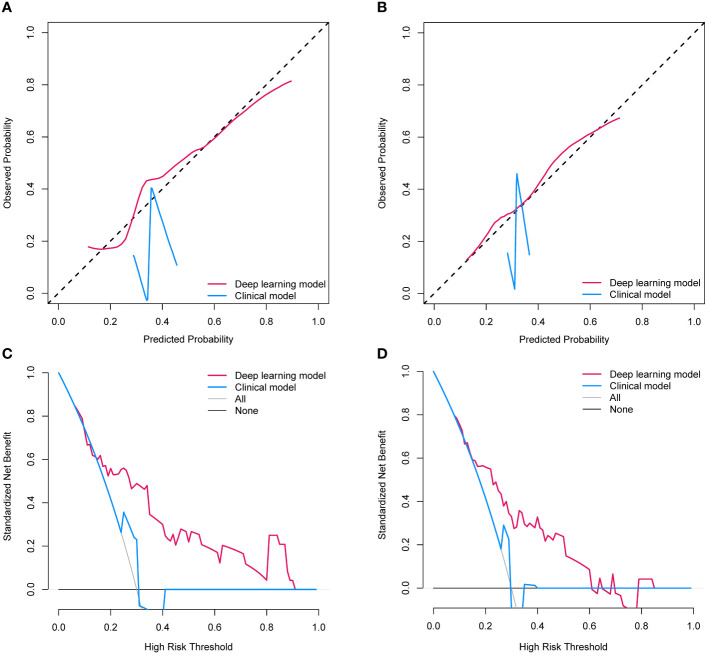
**(A)** The calibration curves of two establish models in the validation set; **(B)** The calibration curves of two establish models in the external cohort; **(C)** The decision curves of two establish models l in the validation set; **(D)** The decision curves of two establish models and clinical model in the external cohort.

To evaluate the clinical usefulness of the deep learning model for pCR prediction, the calibration curve and decision curve analyses were performed, revealing that the deep learning model conferred better predictive performances (**Figures 5A, B**) and higher net benefits (**Figures 5C, D**) than the clinical model in the validation set and external cohort. In addition, **Table 3** shows the positive value of net reclassification improvement (NRI) and integrated discrimination improvement (IDI) of the deep learning model compared to the clinical model. The deep learning model could achieve positive NRI and IDI no matter in the validation set (NRI: 0.222 [95% CI: 0.039, 0.299], p=0.028; IDI: 0.143 [95% CI: 0.059, 0.227], p<0.001) or external cohort (NRI: 0.186 [95% CI: 0.018, 0.391], p=0.023; IDI: 0.128 [95% CI: 0.033, 0.224], p=0.008).

**Table 3:** Net reclassification improvements and integrated discrimination improvements of deep learning model compared to clinical models.

Cohort	NRI (95% CI)	p value	IDI (95% CI)	p value
Validation set	0.222 (0.039-0.299)	0.028	0.143 (0.059-0.227)	<0.001
External cohort	0.186 (0.018-0.391)	0.023	0.128 (0.033-0.224)	0.008

NRI, Net reclassification improvement; IDI, integrated discrimination improvement; CI, confidence interval.

### Biological basis exploration

**Figure 6:**
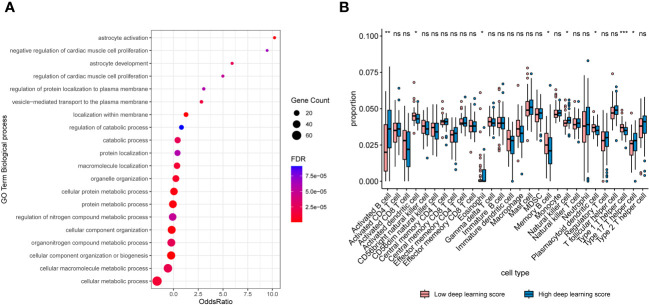
**(A)** Bubble plots exhibiting the top 20 regulated biologic processes among cases with high deep learning scores. **(B)** Boxplots exhibiting proportions of immune infiltrations between patients with high and low scores. *, p < 0.05; **, p < 0.01; ***, p < 0.001. NS, not significant.

As depicted in **Figure 6A**, noticeable distinctions were evident in gene expression patterns among patients with high and low scores. In cases with high deep learning scores, there was a substantial upregulation of pathways associated with cell proliferation and metabolism, including regulation of catabolic process, catabolic process, cellular protein metabolic process, regulation of nitrogen compound metabolic process, organonitrogen compound metabolic process, and fcellular macromolecule metabolic process. Additionally, tumors classified as high-score exhibited increased infiltration of activated B cell, natural killer cell, and type 17 T helper cell when compared to those classified as low-score (**Figure 6B**).

## Discussion

Neoadjuvant immunotherapy, which enhances the likelihood of achieving curative surgery and improves long-term survival compared with traditional neoadjuvant chemotherapy, represents a state-of-the-art approach in treating lung cancer (5–8). However, in spite of significant advancements, a considerable portion of lung cancer patients fail to obtain pCR to neoadjuvant immunotherapy (9). In such cases, there is an urgent need for an effective method to recognize candidates who might potentially benefit from this cutting-edge therapeutic strategy. The present multicenter study developed a deep learning signature for pCR prediction in NSCLC following neoadjuvant immunotherapy and the proposed model achieved AUCs of 0.775 and 0.743 in the validation set and external cohort, respectively.

The assessment of neoadjuvant immunotherapeutic efficacy in NSCLC in a quantitative and efficient manner has been a topic of passionate debate. For a long time, the absolute survival improvement has served as the primary criterion for determining neoadjuvant efficacy since the introduction of neoadjuvant therapy as a systematic approach (24). However, the necessity for long-term follow-up hampers the effectiveness of survival outcomes as the sole endpoint. The recent surge in clinical trials for neoadjuvant therapy, particularly neoadjuvant immunotherapy in the past few years (5–8), has highlighted the need for more effective parameters beyond survival. Pathologic response has emerged to be a robust indicator of prognosis in various solid tumors (25). CheckMate-816 (26), the initial Phase III clinical trial investigating the effectiveness of neoadjuvant chemoimmunotherapy in NSCLC, revealed an enhanced event-free survival within the group of patients who achieved pCR. This outcome underscores that obtaining pCR is indicative of the survival benefits associated with neoadjuvant chemoimmunotherapy. However, the incidence of pCR, as observed in the CheckMate-816 trial, was limited to 24% of patients. In situations like these, it becomes imperative to identify patients who may potentially achieve pCR before initiating the treatment, thus enabling personalized neoadjuvant chemoimmunotherapy for NSCLC.

CT has been a standard instrument in clinical practice for assessing treatment responses in lung cancer. To some extent, CT imaging can evidently depict lesion diameters, which might serve as indicators of tumor burden. Changes of tumor size could dynamically monitor treatment responses. It has been reported that the use of tumor diameters in CT to predict pCR in the context of neoadjuvant chemotherapy and targeted therapy. Nevertheless, when it comes to immunotherapy, a unique treatment approach where drugs indirectly suppress tumor growth through activating the immune system, treatment responses might occur before the gross size of the tumor regresses (27). As a result, radiologic regression might not precisely reflect pathologic regression following neoadjuvant immunotherapy ^7^. In some cases where pCR occurs, the radiological size of the tumor may even appear to increase due to immune cell infiltration. Therefore, relying solely on superficial CT characteristics is insufficient for accurately predicting the pathologic response. Instead, it becomes necessary to extract more deeper radiologic phenotypes to predict pCR following neoadjuvant immunotherapy in the NSCLC patients.

Emergence of radiomics based on deep learning, which holds the advantage to uncover hidden imaging features beyond human visual perception (15), has opened up new possibilities for predicting the effectiveness of neoadjuvant immunotherapy. Deep learning’s application in the analysis of radiomics features in tumors has played an increasingly pivotal role in tumor diagnosis, treatment decision-making, and survival prediction (16–18). Abundant publications have validated the underlying relationships between CT deep phenotypes and immunotherapy efficacy in advanced lung cancer patients treated with ICIs (21, 22, 28). This offered a strong rationale for conducting the present research. In addition, in neoadjuvant settings, prior studies have successfully developed radiomics signatures to estimate the likelihood of pathologic response following neoadjuvant chemotherapy in various tumors, including lung cancer. These models achieved AUCs ranging from 0.63 to 0.73 (19, 20). However, despite these efforts, investigation into the feasibility of using radiomics representations to predict the efficacy of neoadjuvant immunotherapy is limited.

Our study employed a deep learning technique to construct an imaging signature to predict pCR in NSCLC patients undergoing neoadjuvant immunotherapy, the proposed model demonstrated satisfactory efficiency, with the AUC of 0.743 among the multicenter external population. This suggests its potential utility in identifying NSCLC patients who are likely to respond favorably to neoadjuvant immunotherapy. In such instances, our proposed signature harbors the potential to help surgeon optimize the neoadjuvant administration for lung cancer patients. For one thing, if a patient is predicted to have a high deep learning score of pCR, the doctor could administrate further molecular tests to confirm the suitability for neoadjuvant immunotherapy. For another thing, if a patient achieved a low deep learning score of pCR, invasive biopsy procedures and expensive molecular testing could be waived.

However, despite the potential of our proposed signature in recognizing patients sensitive to neoadjuvant chemoimmunotherapy, there are some specific considerations that need to be addressed for applying this signature into the real clinical scenario. The main challenge is that the efficiency was not adequate to serve as a direct determinant of pCR considering our proposed model only achieved AUCs of 0.743-0.775. Nevertheless, neoadjuvant immunotherapy is a newly proposed therapeutic strategy in recent years, artificial intelligence studies on this topic were limited by now. Currently, several studies demonstrated the utility of deep learning algorithm based on sequencing data in prognosis prediction, disease identification, and treatment decision (29–32), which implies the potential of multi-omics data in predicting neoadjuvant chemoimmunotherapy efficiency. We believe that future studies including more data modalities and more advanced algorithms could improve the accuracy of the artificial intelligence model for predicting pCR to neoadjuvant chemoimmunotherapy.

The current study has several limitations which should be noticed. Firstly, given its retrospective nature and the study’s focus on the Chinese population, it is inevitable that the selection bias and discrepancy of pCR proportion might exist. Further larger research with a diverse, multiethnic patient population and a prospective setting are warranted to address these limitations. Secondly, since all patients included in the study were treated after 2019, the correlations between the deep learning score and survival outcomes remains unclear. Therefore, further studies with survival as the endpoint are needed to assess the comprehensive performance of the deep learning signature. Thirdly, the model construction solely relied on CT modality, leaving room for improvement in algorithm precision. Subsequent studies will aim to enhance accuracy by incorporating additional imaging modalities into the deep learning network to predict neoadjuvant immunotherapeutic efficacy more effectively. Finally, high-resolution CT findings is necessary to analyse the subtle images therefore it is difficult to obtain such precise findings using the conventional CT images.

In conclusion, the deep learning model utilizing CT imaging, exhibits a notable capacity for predicting pCR in NSCLC patients undergoing neoadjuvant chemoimmunotherapy. Furthermore, the inherent biological foundation of deep learning prediction appears to have associations with pathways governing cell metabolism and the facilitation of antitumor immune infiltration within the microenvironment.

## Data availability statement

The raw data supporting the conclusions of this article will be made available by the authors, without undue reservation.

## Ethics statement

Written informed consent was obtained from the individual(s) for the publication of any potentially identifiable images or data included in this article.

## Author contributions

WQ: Writing – original draft. CCh: Writing – original draft. QL: Writing – original draft. CCa: Writing – original draft. MG: Writing – original draft. YS: Writing – review & editing. MY: Writing – review & editing. MS: Writing – review & editing.
